# Improving medical students’ learning strategies, management of workload and wellbeing: a mixed methods case study in undergraduate medical education

**DOI:** 10.1186/s12909-025-07118-6

**Published:** 2025-04-24

**Authors:** Christopher-James Harvey, Kathleen E. Leedham-Green, Cristina Koppel, Arti Maini, Susan F. Smith, Mary J. Morrell, Michael Emerson

**Affiliations:** 1 School of Public Health, Imperial College London, London, W127 TQ UK; 2 Medical Education Research Unit, Imperial College London, London, UK; 3 Chelsea and Westminster Hospital, London, London, UK; 4 National Heart and Lung Institute Imperial College London, London, UK; 5 Pears Cumbria School of Medicine, National Heart and Lung Institute Imperial College London, London, UK

**Keywords:** Mindset, Self-efficacy, Study skills, Revision, Well-being, Tutoring

## Abstract

**Background:**

The transition from secondary education to university challenges students’ learning strategies and academic performance, especially in self-directed, problem-based environments like medical school. Passive study methods often fail, while evidence-based strategies like retrieval practice, active learning, and growth mindset foster success. We evaluate a novel academic support programme (Academic Tutoring- (AT)) to enhance study skills, feedback use, and self-directed learning.

**Methods:**

We developed and implemented AT for 1st year medical students, informed by the psychology of learning and behaviour change, AT aimed to support the development of self-efficacy and effective learning strategies during the transition into university. The programme involved meeting an Academic Tutor one-to-one once per term, and also as a group once per term. Academic Tutors engaged students in learner-centred conversations on study skills and professional development plus their wellbeing and welfare. A Likert questionnaire was designed to measure students’ responses to the experiences and perceived outcomes of AT. We also measured self-efficacy and mindset. Qualitative data was gathered through open-ended response items. Demographic and socioeconomic data was also gathered.

**Results:**

AT positively impacted time-management and learning strategies. ‘Learning from successes and failures’ and ‘thinking how to achieve goals’ were associated with a growth mindset. All outcome measures were associated with self-efficacy. We noted that students from a widening participation (WP) background tended to show higher growth mindset relative to those from a non-WP background (r = -0.223, p = 0.08) and female students reported higher engagement with the programme (r-0.294, *p* < 0.001). Students reported changes in behaviours and attitudes, and improved wellbeing.

**Conclusions:**

Providing medical students with the tools to change their approach to work and revision can improve subjective reports of time management, implementation of successful learning strategies and wellbeing. Successful outcomes were associated with self-efficacy and mindset. These are modifiable constructs, and this work suggests that focussing conversations on self-efficacy and mindset may be beneficial for supporting positive behaviour change.

**Supplementary Information:**

The online version contains supplementary material available at 10.1186/s12909-025-07118-6.

## Background

### The problem

The transition out of secondary education marks the onset of a new developmental phase. Riordan and Carey [1], with reference to Arnett’s framework of emerging adulthood [2], suggest it is a period of ‘in between’ – not quite an adult and not quite an adolescent. This ontological shift has been associated with more stress, and uncertainty. In our medical students at Imperial College London we have observed struggles with ongoing attainment—an unexpected shift from achieving to a high level academically relative to peers, to middle or under-performing in a relatively-large medical school despite reported effort.

The learning strategies that these students employed successfully at school do not appear to be effective in their new learning environment. Like many higher education programmes, our medical degree course has a greater emphasis on self-directed study, including engagement with flipped or blended elements, problem-based and team-based learning, plus assessments that require integration and application of knowledge from multiple modules. There is evidence to suggest that students are not always ready to engage actively on entering university [3].

The literature on successful learning highlights that passive study strategies, such as re-reading, watching lectures, highlighting and copying out notes, may feel productive but are in fact relatively ineffective [4]. Brown et al. suggest that effective learning should feel like hard work: anything that feels easy is probably unproductive and needlessly time consuming. They suggest that learners are poor judges of what works, and should rely on evidence rather than intuition, as many strategies that feel productive may simply be creating the ‘illusion of learning’. The strategies they suggest include:

- retrieval practice, for example through self-testing
- spaced repetition and revisiting topics regularly, for example through flashcards
- interleaving topics, rather than studying subjects as a block
- strategies to optimize attention and focus, for example using timers and minimizing distractions
- structured note-taking, where learners actively organize rather than simply record what they are hearing
- active problem solving rather than looking up solutions, thereby learning underlying principles rather than specifics
- self-monitoring and responding to feedback, and adapting learning strategies accordingly
- building on foundational knowledge through mental models and mind maps, and
- linking abstract knowledge to future practice through real-world examples and narratives.

We also explored the literature on mindset, which suggests that a learner’s approach to feedback and risk is also a significant predictor of educational outcome [5]. Dweck suggests that some students avoid feedback, seeing criticism as failure and an indication of their innate ability, whereas more successful students embrace feedback as an opportunity for growth. A recent meta-analysis suggests that growth mindset can be nurtured through high quality feedback processes and growth-oriented conversations, and that it is a replicable and generalisable predictor of academic attainment, particularly among people facing difficulties and academic set-backs [6].

Self-efficacy is another attribute that has been shown to be positively associated with academic attainment [7]. Albert Bandura defines self-efficacy as belief in one’s capabilities to organize and execute a course of action [8]. Its importance for academic achievement relates to how soon one gives up, and to what extent one is prepared to keep working on a problem until it is solved. Specific strategies to nurture self-efficacy include goal setting, feedback that enhances self-calibration, and peer modelling [9].

None of these learning theories or practices are new, however their systematic application to medical education on a cohort level is. We therefore aimed to create and evaluate a novel Academic Support Programme (AT) to help our first-year medical students flourish, by building efficient and effective study skills. The programme also aimed to support students in acquiring skills needed for becoming capable, self-directed learners who actively seek and respond positively to feedback. This is in line with contemporary theories of education which aim to maximise engagement, rather than minimising disengagement (or providing support only to those who struggle) [10].

### The intervention

The implementation of the programme was informed by the psychology and neuroscience of behaviour change. Learners need to feel motivated to try new learning behaviours, they need to understand and feel capable of enacting those behaviours, and they need social cues and physical opportunities that encourage those new behaviours within their learning environment [11]. This follows a COM-B approach to behaviour change, encapsulating a holistic view of behaviour change theory [12]

The intervention represents a shift from the predominant and traditional ‘personal’ tutoring model. In the traditional model, the tutor is primarily there for welfare-support- ‘*in loco parentis’*. Our model encourages tutors—now termed *Academic Tutor*—to actively engage students in student-led conversations around their study skills and professional development [13], as well as engaging in conversations around wellbeing and welfare. The aim of introducing a focus on the development of key study skills was to reduce stress and anxiety during a major transitional period by promoting engagement and self-efficacy. One of the aims of the *Academic Tutor* was to move away from the tutor as ‘fixer’ to the tutor as ‘facilitator’, so that students felt encouraged to take ownership and act on their own personalised goals and solutions.

To prime students’ study skills and knowledge of evidence-based approaches to learning, we introduced a lecture on the “The Science of Successful Learning” in their first week at university, which was also shared across our network of *Academic Tutors*.

To support students with building motivation for change, we provided training for our tutors in coaching skills for learner-centred conversations. Tutors were introduced to the GROW coaching model [14] which provides a structured framework for non-judgemental, non-directive, solution-oriented conversations to support goal setting and action planning. [14, 15]; A version of this training is now available online [16]. Students had termly one-to-one tutorials where they were invited to set their own study goals, to devise action plans for achieving those goals, and to articulate strategies for ongoing self-monitoring and problem solving.

Finally, we addressed the learning environment by setting up a series of four structured group tutorials to build a community of practice between learners where students were invited to share and discuss learning strategies [17]. These group tutorials covered topics such as active learning, time management, engagement with feedback, exam preparation and professionalism as well as welfare and wellbeing topics. Students could request additional meetings and access a range of welfare and support initiatives. Tutors were supported through group meetings, training and ad hoc support from the Head of Academic Study Skills and a Senior Welfare Tutor, who focussed solely on welfare support.

## Methods

### Aims

We aimed to evaluate both our outcomes and processes, exploring for whom this programme worked, and why. We framed these aims in the 4 questions below:

- What educational value was created through our revised academic support practices?
- Who did the programme work for and why?
- What were the experiences of students that engaged with the programme, and how did this vary across subgroups?
- What suggestions for improvement did students have?

### Context

The research involved Year 1 undergraduate medical students and was conducted at Imperial College School of Medicine. Imperial College London is a highly ranked university which contributes to a motivated, competitive intake [18].

### Research design

Our research is situated in a critical realist research paradigm [19]. This necessitates the following assumptions:

- we accept causal relationships between phenomena within a real physical world, however,
- we argue that experiences are necessarily subjective;
- outcomes are influenced by sociocultural factors such as identity and social capital;
- data needs to be interpreted within its context.

We adopted a mixed methods evaluation involving a simultaneous nested design survey [20].

### Research team & reflexivity

The research team included a full-time educational researcher (KLG) who was not involved in planning or delivering AT; a professor of medical education, and *Academic Tutor* (SS); a research-psychologist and *Academic Tutor* with statistical training (CJH); a clinical neuroscientist and *Academic Tutor* (CK); a clinician, accredited coach and *Academic Tutor* (AM), a research professor and head of year (MM) and a research professor and course lead for the *Academic Tutoring* programme (ME). All authors were involved in designing the survey and in the data analysis apart from CJH who contributed to analysis and write-up. KLG and CK coded the data which was audited by SS and worked together to construct themes and categories. CJH and KLG did the statistical analysis. ME designed and ran the tutoring programme. AM designed and led the coaching training for Academic Tutors. All were involved in the overall interpretation and discussion. Positionality was mitigated through reflexive discussions and auditing to ensure all views, positive and negative, were included.

### Participants

All 365 first-year medical students were invited to participate via a lecture shout-out and newsletter.

### Data generation

The survey design began with a modified Delphi technique, where all 17 Academic Tutors- including those involved in this paper- were invited to suggest research questions which were then voted on. The most popular questions were honed by the research team to enhance clarity and reduce redundancy. The survey included four questions relating to experiences and four questions relating to outcomes. This follows a model often used in medical research where Patient Reported Experience Measures (PREMS) and Patient Reported Outcome Measures (PROMS) are developed to understand fully the impact of the interventions on the user. This survey can be seen in appendix 1. Two validated scales were used to assess self-efficacy [21] and mindset [22]. The survey was hosted on Qualtrics (Qualtrics software, Version August 2019 of Qualtrics. Copyright © 2019 Qualtrics), and piloted and further tested by two teaching fellows and two medical students who collaboratively improved its clarity, acceptability and usability. Agree/disagree sliders were chosen with a neutral starting point as these have been shown to perform similarly to Likert scales [23] and were preferred by our testers. All questions were positively framed.

**Fig. 1:**
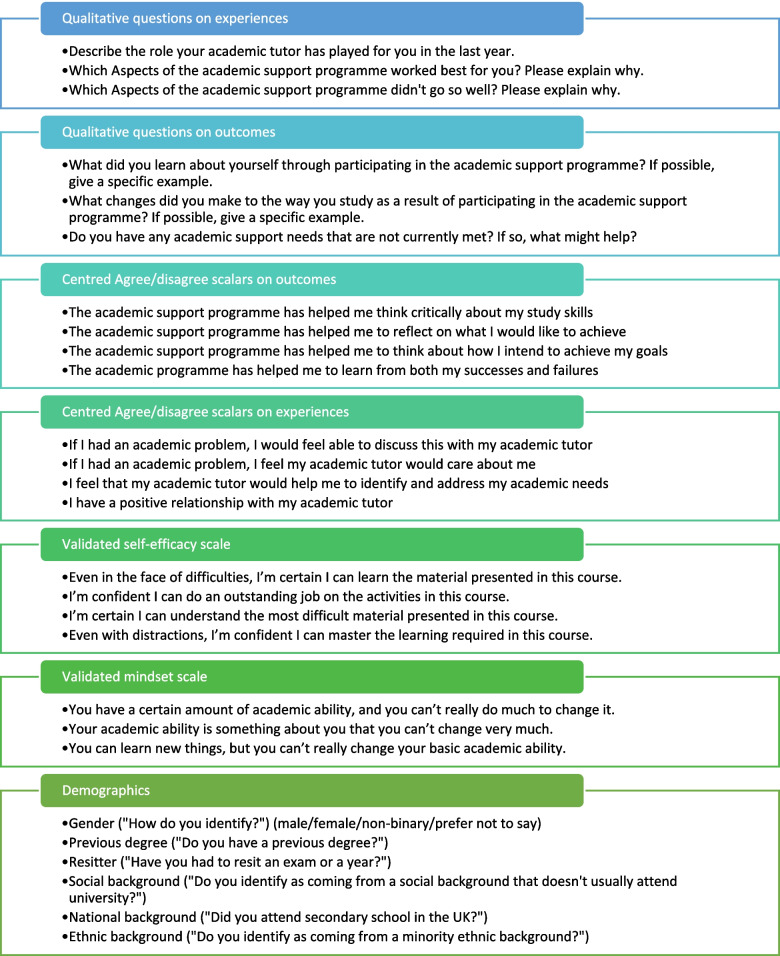
Each quantitative section ended with a text box inviting participants to explain why they had answered as they did. Agree/disagree questions were scalar sliders, centred at a neutral point. Demographic options were yes/no/prefer not to say, except for gender which was male/female/non-binary/prefer not to say

Nominal data were collected relating to ethnicity, nationality, social background, gender, age, previous degree, and whether students had had to resit a year or exam. The survey is summarized in Fig. 1.

### Data analysis

## Qualitative analysis

Qualitative data relating to processes were coded inductively using a consensual process [24]. Two researchers, KLG and CK, coded a sample of responses together, then worked concurrently to code a random selection of responses, conferring with each other where meaning was potentially ambiguous or coding decisions needed to be made. They worked together to group similar content into themes and finally into higher level categories. Saturation was determined when all new content was being coded into pre-existing themes which was achieved after 40 responses; 60 responses were fully coded. All the remaining responses were read to ensure no minor themes had been missed and the most illuminating quotes were identified from the full set. Themes were audited by SS.

**Fig. 2:**
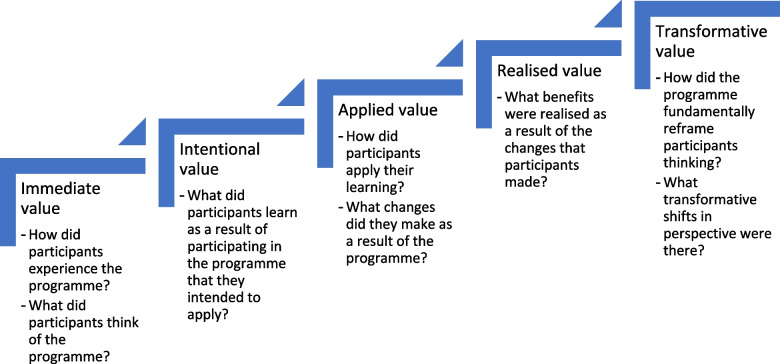
Adapted from the Wenger-Trayner framework for categorising educational value in communities of practice

Themes relating to outcomes were placed into prescriptive categories relating to the Wenger Trayner levels of educational value within communities of practice [25]. This framework allowed the researchers to conceptualise educational value as immediate, intentional, applied, realised or transformative and is illustrated in Fig. 2.

Quantitative analysis and mixed methods integration.

We conducted an exploratory data analysis which aimed to inductively identify patterns and associations within the data, rather than to prove a priori hypotheses [26]. For this reason, we were not overly concerned with type 1 errors and have not conducted corrections for multiple tests of significance [27].

We conducted a series of Spearman’s non-parametric bivariate analyses to explore whether our eight sliders relating to the impacts and experiences of the programme were associated with higher mindset or self-efficacy scores. Where significant correlations were found we went back to the qualitative data to illuminate those phenomena further.

We explored the relationship between demographics and responses to our experience and outcomes measures and also mindset and self-efficacy scales. We then analysed qualitative comments and contextual factors to hypothesise about the underlying reasons for any observed differences.

## Results

### Demographics

We received 141 responses (38.6% of 365). Respondent demographics are listed in Table 1.

**Table 1:** Respondent Characteristics. Total *n* = 141 except for ‘Attended Secondary school in the UK’; ‘Do you have a previous degree’, and ‘Do you identify as a widening participation student (from a social background where few people go to university)?’ where *n* = 140 due to missing data

What is your age?	Median	19
	Range	18–25
		N (%)
What gender do you identify as?	Male	62 (43.97)
	Female	78 (55.32)
	Non-binary/prefer not to say	1 (0.01)
Attended secondary school in the UK	Yes	113 (80.71)
	No	27 (19.29)
Do you identify as minority ethnic?	Yes	67 (47.52)
	No	72(51.06)
	Prefer not to say	2 (1.4)
Do you have a disability requiring reasonable adjustments?	Yes	6 (4.25)
	No	135 (95.74)
Have you retaken a year?	Yes	4 (2.83)
	No	137(97.16)
Have you re-sat an exam?	Yes	5 (3.55)
	No	136(96.45)
Do you have a previous degree?	Yes	9 (6.43)
	No	131 (93.57)
Do you identify as a widening participation student (from a social background where few people go to university)?	Yes	25 (17.86)
	No	115 (82.14)

### Exploration of demographics and experience of the programme

#### Gender

There was a positive correlation between being female and higher Likert responses to ‘The AT has helped me think critically about my study skills’ (*r* = 0.19, *p* = 0.02); and ‘The AT has helped me to think about how I intend to achieve my goals’(*r*- 0.294, *p* < 0.001). The reason for stronger reported outcomes for females in these domains was not immediately apparent from qualitative responses, however comments from female participants highlighted that some may not have been asked to think critically about their own goals before:

“It made me think more critically about the best way to achieve my goals as I hadn't thought about it much before.” R2 (female)

“The programme has helped me critically think about my study skills because it was something I had never really put any effort into before.” R131 (female)

#### Graduate entry

Being a graduate student was associated negatively with ‘The AT has helped me learn from both my successes and failures’ and positively with ‘If I had an academic problem, I would feel able to discuss this with my tutor’ and ‘I have positive relationship with my tutor’ (*r* = 0.207, 0.1891, 0266 respectively, *p* > 0.05). Being a graduate student was also associated with higher self-efficacy score (*r* = − 0.17, *p* = 0.04).

There were no explanatory comments, however, a contextual analysis revealed all graduate students had the same highly experienced *Academic Tutor*, and as graduate entry, when offered, was so competitive at this institution (1:15 offer ratio), these students may have already developed self-efficacy and/or have relatively limited experience of academic failure.

#### Widening participation

Identifying as a student from a ‘widening participation’ (WP) background, relating to being from a disadvantaged and/or under-represented group was associated with a moderately higher mindset score (r = − 0.223, p = 0.08). Qualitative comments suggested that these students’ growth mindsets preceded the course:

“I already knew how to learn from my successes & failures” R60.

Some WP students expressed positive affirmations and statements of self-belief that read like personal mantras:

“Without believing I can do it I'll never do it.” R60

#### Nationality and ethnicity

The eight experience and outcome measures were reported similarly by students that went to secondary school in the UK or abroad, and for students who identified as majority or minority ethnic. Mindset and self-efficacy scores were not significantly different across these groupings.

### Exploration of mindset, self-efficacy, and experience of the programme

**Fig. 3:**
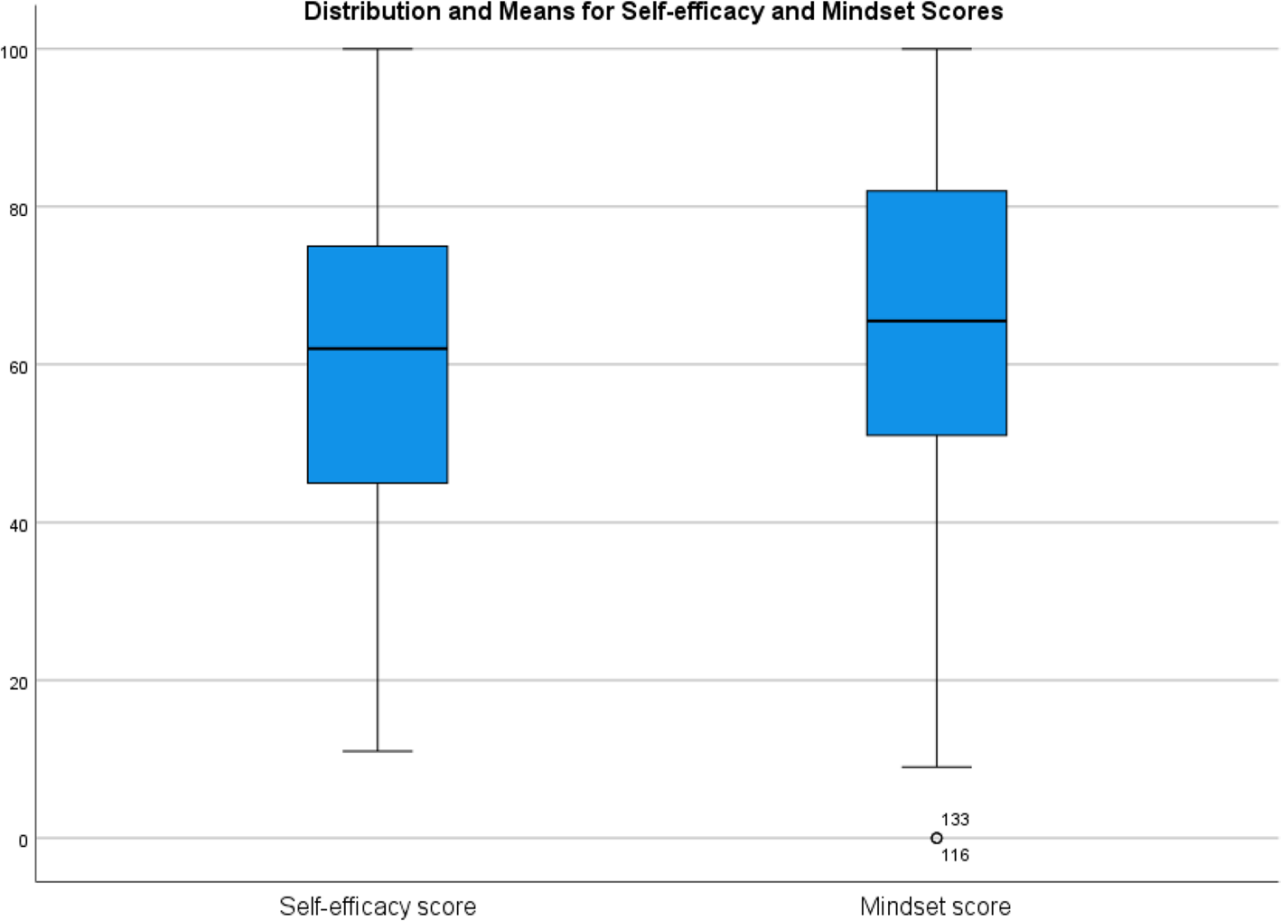
Boxplots showing spread of self-efficacy and Mindset responses

Descriptive data for mindset and self-efficacy are displayed in Fig. 3.

There was no correlation between mindset and self-efficacy (*r* = 0.12, *p* = 0.18) confirming they are separate constructs. Table 2 shows the relationships between self-efficacy/mindset and the Likert responses. The Likert scores relating to learning from both successes and failures and thinking how to achieve goals were weakly associated with mindset. All Likert scores relating to outcomes, and three out of four Likert scores relating to experiences (excluding having a positive relationship with their *Academic Tutor*) were moderately associated with self-efficacy.

**Table 2:** Correlation coefficients and *p*-values for mindset/self-efficacy against Likert responses on programme experiences and outcomes

		The AT has helped me think critically about my study skills	The AT has helped me to reflect on what I would like to achieve	The AT has helped me to think about how I intend to achieve my goals	The AT has helped me to learn from both my successes and failures	If I had an academic problem, I would feel able to discuss this with my academic tutor	If I had an academic problem, I feel my academic tutor would care about me	I feel that my academic tutor would help me to identify and address my academic needs	I have a positive relationship with my academic tutor
Self-efficacy score	r	.290^**^	.304^**^	.291^**^	.359^**^	.257^**^	.317^**^	.325^**^	.156
	*P* value	** <.001**	** <.001**	** <.001**	** <.001**	**.002**	** <.001**	** <.001**	.072
	N	136	135	136	135	137	136	135	133
Mindset score	r	.078	.146	.175^*^	.173^*^	.048	.152	.089	.192
	*P* value	.367	.089	**.041**	**.045**	.581	.077	.305	**.027**
	N	137	136	137	136	136	136	134	132

^**^indicates a moderate association; ^*^Indicates a weak association; bold indicates *p* < 0.05

### Thematic analysis

Analysis of the student responses to the opened ended survey questions led to identification of a number of common themes which are summarised in broad groupings relating to immediate educational value (Table 3) and transformative educational value (Table 4).

**Table 3:** Themes relating to immediate and intentional educational value

**Immediate educational value (experiences, reactions)**	
**Evaluative reactions**	**Experiences of coaching**
- Group tutorials	- Identifying what matters
◦ Good for sharing knowledge	- Goal setting
◦ Bad for personalised approaches	- Action planning
◦ Some groups more functional than others	- Learner-centred conversations
◦ Time consuming	- Strengths-based approach
◦ Reassuring	- Student as resourceful
- Individual tutorials ◦ Good for personal issues/shy	- Self-driven solutions
◦ Dependent on tutor qualities	
◦ Relationships matter	
◦ Meaningful	
- Academic skills lectures	**Experiences of academic tutoring**
◦ Good for knowledge gain	- Learning about strategies for learning
◦ Time efficient	◦ New vocabulary
◦ Useful vs not needed	- Reflection on current strategies
	- Sharing and discussing strategies
	- Trying new strategies out
	- Seeing what works personally
	- Trying to embed/sustain new
	- strategies
**Affective reactions**	**Experiences with tutors**
- Positive	- Tutor qualities
◦ Relief	◦ Respect
◦ Reassurance	◦ Curiosity
◦ Gratitude	◦ Caring
◦ Hope	◦ Approachable
- Negative	◦ Intimidating
◦ Disengaged	- Tutor approaches
Didn’t need help	◦ Caring vs tick-box approaches
◦ Frustrated	◦ Directive vs facilitative approaches
Didn’t get help	◦ Agenda-based vs agenda-free approaches
Time/travel commitment too high	◦ Relationship building
**Intentional education value (intentions to change)**	
- Difficulty initiating change	
- Difficulty sustaining change	
- Ongoing intention to change

**Table 4:** Themes relating to applied, realised and transformative educational value

**Applied educational value (applied learning)**	
**Active learning strategies**	**Time management strategies**
- Spaced repetition	- Pomodoro technique
- Interleaving topics	- On-the-go revision
- Interlinking topics	**Note taking strategies**
- Active recall	- Structured notes
◦ Self-testing	- Mindmaps
◦ Flashcards	
**Concentration strategies**	**What strategies did they stop?**
- Adaptations to environment	- Passive strategies
- Breaks	- Underlining
- Exercise	- Copying out
- Reducing distractions	
- Timeboxing	
**Realised value (impacts of changes)**	**Reframing value (transformative impacts)**
**Improved learning**	**Self-efficacy**
- Improved memorisation	- Confidence problem solving
- Deeper understanding	- Self-confidence
**Improved time-management**	**Meta-cognition**
- More time for socialising	- Self-awareness
- More time for wellbeing	- Critical reflection
- Getting through the content	- Self-regulation
**Improved wellbeing**	**Mindset**
- Awareness and prioritisation of mental health	- Willingness to seek help
- Reduced overwhelm	- Willingness to seek feedback
- Personal growth	- Confidence in ability to succeed
- Insights into ‘what matters’	- Motivation to succeed
	- Willingness to try new things

In line with the Wenger-Trayner framework, we present the subthemes related to immediate and intentional educational value and will then discuss subthemes relating to applied, realised and transformative educational value.

### Immediate educational value: how was the programme experienced?

#### Experiences related to coaching approaches

Participants appreciated being treated as autonomous individuals, with respect and curiosity, but without judgement, rebalancing the power dynamic and creating the conditions for honest conversations.

“My academic tutor is really caring and treats me like a peer, not a student, so I feel really comfortable discussing my work ethic/ethos and goals.” R100

Participants appreciated personalised approaches that related to the learner’s agenda:

“The sessions were tailored exactly to my needs and what I wanted to talk about, so I was able to make the most out of these. It helped to have a personal one-to-one session to really get to the roots of my problems and what I wanted to talk about.” R107

Participants appreciated having the opportunity to articulate and reflect on their own goals in order to identify solutions:

“You sometimes need to say things out loud to someone to put things in perspective and realise what you need.” R7

Participants valued a caring, relational approach to conversations, enabling identification of personal wellbeing goals:

“[My tutor] helped me realise that spending time taking care of myself is very important. [They] also checked back with me the next time we met which meant a lot to me. After the break I felt much better and have since then felt great about my mental health and balancing personal time with schoolwork.” R19

Participants appreciated a non-directive approach that moved away from advice giving to generating a space for the students to identify their own solutions:

“She didn't tell me what she would do in my place, but kind of helped me put things in perspective to realise on my own.” R7

#### Experiences relating to study skills

Participants appreciated learning about evidence-based study strategies, but also being encouraged to try things out, self-monitor and see what worked for themselves:

“I knew that my methods of revision weren't ideal, but I was struggling to figure out how I should change them. My tutor guided me to think about better study methods which worked best for me.” R133

“The time management exercise (keeping a diary of how many hours per week are spent doing different activities) was also really useful in identifying where I was wasting time and allowed me to improve my productivity.” R81

“More that it's the process of discovering your ability and skills. The way you learn as opposed to shoehorn yourself into a specific archetype.” R17

“I definitely think I have started evaluating my own study techniques more as a result of the academic tutor programme. I spend time thinking about which way works best for me in terms of learning.” R42

#### Experiences relating to wellbeing

In addition to discussions relating to study skills, tutors encouraged personal development and provided practical and emotional support. Some participants appreciated a structured approach to ‘checking in’ across a range of domains (housing, finances, health, careers, friends & family, harassment) during one-to-one sessions. Where this was not present some had difficulty broaching personal topics.

“I personally feel the 1-to- 1 sessions could be improved by having a prior agenda sort of set to base the session around. While they were a good check-up it often resulted in"so is there anything else you wanted to say or add".” R17

Others wanted less structure and more personalised approaches.

“It's like they had a template to follow and nothing was PERSONAL. I wish that there was more emphasis on the personal side of the programme.” R134

#### Reactions to the different programmatic components

Participants expressed differing personal preferences for the three different components of the programme. Some preferred the study skills lectures which were described as more time efficient for knowledge transfer; some preferred the group sessions which were described as more engaging and helpful for sharing, comparing, and critiquing learning strategies; and others the one-to-one tutorials which were described as more tailored and personal. Some had already developed effective learning strategies and did not feel they needed the programme.

“Talking to other people in the group made me realise different things about the learning style, also reassured me about how to revise.” R29

“I already had a study technique that worked for me at the beginning of the year and… sessions about how to find your preferred working technique felt a bit useless. I understand that this is important for some people but it's difficult to cater for the needs of everyone in a single group session… In the one-to-one sessions I felt much more comfortable to talk about the things that worried me and open up to my tutor about the things I needed help with.” R7

“The academic tutorials were good, but they weren't as informative about study skills as the lectures were.” R23

Some articulated changing preferences over the course of the academic year, wanting an emphasis on study skills and relationship building at the start of the year, moving towards more flexible, personalised approaches during exam season.

#### Affective reaction

Some expressed surprise at not having thought about their study skills before and gratitude for dedicated time and space to contemplate this. The programme was described as meaningful and important and elicited expressions of appreciation, and feelings of reassurance and hope.

“[The] tips on repetitive active learning were great. Also, the use of a 2nd year who failed his mock but came top in actual exam using active learning gave hope to people who felt a bit overwhelmed without making people complacent.” R100

#### Intentional value: what did students learn but not use?

Some reflected on their learning strategies and recognised the value of trying new approaches but found change difficult to initiate or maintain. Some appeared stuck on the cusp of moving from passive to active learner:

“[I learned] about how to plan my time more efficiently, although I found it hard to implement... I didn't [change] very much, although I tried to focus less on passive revision methods… I would prefer to have [the group tutorials] as information sessions rather than so interactive.” R2

#### Applied Educational Value: how did students apply their learning? Applied, Realised and Transformative Educational Value

One hundred twenty-four out of 141 participants described changes they had made to their study habits because of this programme. Changes included more self-testing and self-monitoring, stopping or reducing passive strategies such as underlining and copying out, spaced repetition and interleaving between topics, linking and integrating across topics, preparing before live teaching sessions, changing the way they took notes including mind mapping, changing their working environment or place of study, earlier revision planning, revising “on the go” through podcasts and flashcards, and strategies for maintaining attention and motivation such as timers, chunking, exercise breaks, and switching off social media. Participants described using a variety of digital and paper flashcards which they either created, bought or shared. Some had gone on to read the recommended text “Make It Stick” [4].

“My revision was very passive and during my A-Levels I revised by just reading over notes and directly attempting exam questions, however, because of the large amount of information that we have to learn as medical students, this simply wasn't working anymore, especially when trying to recall very minute pieces of information. Now I have changed my revision technique to be more active by using flashcards, brainstorms and then supplementing with exam questions.” R67

### Realised value: what were the benefits of these new ways of working?

Participants described improved learning, time-management, and wellbeing.

#### Improved learning

Many were effusive about the educational value of their new active study techniques. Some expressed dismay at the time they had wasted on ineffective learning and revising the wrong way. They expressed a new understanding and surprise at the range of methods available and their varying success. They noted a deeper understanding of the material, better retention of information, and improved memory in general. Flashcards were described as effective for topics requiring broad memorisation such as anatomy. One described feeling empowered to stop using flashcards for topics where they were less effective. Some described better self-regulation through comparing and discussing strategies with peers. Participants frequently reported struggling to change their technique, but once engaged, found it worth the effort. Overall, they expressed delight and some relief in having found a technique that works for them.

“Although I was resistant to try the techniques suggested at first, I feel as though actually I have found the academic support system to be very helpful in terms of my learning ability… I found that once I began to engage … I started to notice an improvement in my ability to retain information.” R8

“I was quite scared about changing my study method. I learned that my ability to learn is actually a lot more flexible than I thought. I also learned that the way I was studying previously was probably not very effective. I [recently] learned that my current study method was effective after discussing successful exam results as a direct consequence of my [new ways of] studying.” R52

#### Improved time management

Participants described improved time-management, with one surprising themselves by finishing their revision on time. Chunking strategies such as the Pomodoro technique [28] (where the learner works for a pre-set amount of time, breaks for a pre-set amount of time, and takes longer breaks after every 3rd round of work) were described as helpful for getting through the work. On-the-go revision helped some free up time for socialisation and wellbeing activities.

#### Improved wellbeing

Improved self-care was a recurring theme. Students reported improved awareness and prioritisation of their mental health. Some reported feeling happier with their work ethic. Students linked improvements to wellbeing with the coaching approaches used during their one-to-one meetings: having the space to reflect on their own goals gave clarity, reduced overwhelm, improved prioritising skills, supported personal growth, and gave insight into what they wanted to achieve.

“In our 1–2- 1 meeting… I realised that my technique of revision was not appropriate for medical school and that my day-to-day activities could impact my ability to learn like sleep schedule and eating. I… arranged myself to get more sleep to be able to attend lectures and focus more. My tutor helped me seek the counselling and guidance I needed to be able to focus… I've been able to think critically about my studies due to the introduction of the idea of active recall and spaced repetition... Academic ability is something that can be improved and sharpened with focus and time.” R38

“The academic support programme… has made the prospect of being faced with a lot of new information to learn less daunting.” R8.

Participants described positive impacts of evaluating their wellbeing and needs. Mental health improvements were mentioned by some.

### Transformative value: in what ways were students’ perspectives reframed?

There were multiple examples of transformative learning, which had repercussions beyond the programme. Transformative perspective shifts included changes to self-confidence, self-awareness, help-seeking behaviours, critical reflection, motivation to succeed, self-efficacy/confidence problem-solving, mindset, engagement with feedback, self-discovery, willingness to try new things, self-regulation, and adaptability. Participants frequently commented on increased introspection, and drew attention to becoming more reflective, meta-cognitive learners, questioning themselves, their thinking, and the thinking of others.

“I learned that I am more adaptable, and I am more capable of taking on challenges than I realised.” R105

Students openly identified the issues they were experiencing and accepted that if things were not working for them, they could take steps to change things or explore solutions and seek support through discussions with their tutors and peers.

“Academic ability is not a constant, it is fluid. If I'm not happy about my academic ability, I will work my guts off to change it and become better than I am.” R23

## Discussion

We were interested in the impact of a new academic support programme on student study-skills behaviour. This new approach programme adopted a coaching approach, aiming to develop self-efficacy and growth mindset. We were interested in who this programme worked for, and why. We have shown that the programme has a positive impact on time-management and wellbeing. We also demonstrated differences according to social background and demographics, whereby students from a WP background tended to show higher mindset relative to those from a non-WP background; graduate students tended to show increased self-efficacy, and females report higher engagement with the programme. We will discuss each in turn.

Other work has demonstrated that time management improves self-efficacy, creating positive self-regard. [29]. Good time management has also been associated with handling transition better [30], and has been suggested as a target skill for promoting self-care in medical staff [31]. Our findings support the idea that these are mutually reinforcing outcomes, and that good time keeping may be a key variable in promoting learners’ wellbeing.

The existing literature suggests that students from disadvantaged backgrounds tend to be less likely to deploy a growth mindset and often benefit from growth mindset training [6, 32]. Our finding that WP students report growth mindset attitudes, attributed to factors prior to the intervention is therefore unexpected. Other work has found that growth mindset is positively correlated with ‘grit’ – how able a person is to maintain motivation towards a long-term goal [33, 34]. Growth mindset and ‘grit’ have been suggested as exclusive but mutually reinforcing constructs [35]. Getting into a top tier medical school such as Imperial College London is challenging, and we hypothesise that students at Imperial who are from a WP background may be a self-selected cohort for growth mindset (and ‘grit’). The issue of WP and participation in HE is complex. It has been suggested that deciding to participate in HE is hampered by economics, but also more complex interactions between sense of identity, belonging and social-context. Taken together this could mean a more determined approach is taken by WP students [36, 37]. This presents an avenue for further research.

The relationship between self-efficacy and outcomes is consistently more reliable, and stronger relative to the relationship between mindset and tutoring outcomes. Where mindset does correlate with outcomes, it does so with outcomes associated with goal setting and processing success and failure. This is aligned with our approach to promoting growth mindset which sets discussions around expectation management, goal setting and processing failure whereby we encourage students to view failure as a learning opportunity rather than an indictment of their innate capabilities. The correlation between mindset and tutor relationship may reflect the fact that those who endorse growth mindset may find these conversations more comfortable and are therefore more likely to have a positive experience. This is speculative but may present an avenue of research on the importance of easing students into growth mindset conversations. Babenko et.al. discuss avoidance goals- the need to avoid performing more poorly than others. This approach has been posited to manifest as withdrawal as a form of self-protection. It is also associated with perfectionism- all of which have deleterious impacts on academic and wellbeing outcomes [38]. The finding that self-efficacy does not relate to relationship with your Academic Tutor may reflect a perception of less need as those high on self-efficacy have a stronger sense of their ability to achieve the task in front of them. Taken together this could suggest that self-efficacy has greater value in promoting success. Future work should look at the role self-efficacy plays in engagement with support services, and response to failure particularly where it exists alongside a fixed mindset.

We note that those identifying as female report better engagement with the programme. We can consider this through a self-determination theory (SDT) lens [39]. SDT, in summary, aims to understand the social constructs which bolster innate motivation. There are 3 pillars contributing to maximal motivation (and wellbeing)- competence; autonomy, and relatedness. If these needs are met, self-motivation will be maximised and the desired learning internalised. Gender differences in extrinsic motivation have been demonstrated elsewhere and have been related to academic self-efficacy. This might drive engagement with a programme of academic support [40]. A student centred, conversational approach – versus a traditional, more directive approach, may encourage a dynamic that is more reflective and therapeutic. This may be perceived more positively by females [41]. More generally, it is established that men, from adolescence onwards, tend to disengage from health and support systems [42], and show lower levels of educational engagement at University [43].

The Likert results suggest that the student-tutor dynamic plays an important role in how a student responds to the programme. This may be similar to the therapist-effect you see in psychological interventions in certain settings, where therapists have systematic outcomes [44]. This may highlight the importance of rapport building between the student and their Academic Tutor.

## Conclusions

AT has been shown to elicit changes in student behaviours related to their study skills. Students also reported improved time management and wellbeing. This therefore may represent an approach to addressing the problem of poorer mental health outcomes in medical students, relative to other student groups. We do not know from this project how changed academic behaviour translates into academic and welfare outcomes. The delivery of practically orientated content in group sessions, and an individualised approach in one-to-one conversations was highlighted as a key strength. It was evident that tailored coaching approaches in Academic Tutor conversations enabled students to reflect on the issues they were bringing, and to develop personalised solutions. When suggesting improvements to our programme, students expressed diverse and often conflicting views, suggesting highly personalised views on Academic Tutoring. This also implies that a mixed method approach may be necessary to ensure breadth of engagement.

We focussed part of our evaluation on self-efficacy and mindset as these are modifiable constructs associated with success. Engagement with the programme and experience of the programme were indeed associated with self-efficacy and mindset, but it is not possible to infer causation, and we highlight that these constructs are also impacted by an individual’s social history. Future work should explore the effect of the programme over time on self-efficacy and mindset.

In conclusion, there are numerous academic and wellbeing benefits to students if conventional welfare-focussed personal tuition is extended to the Academic Tutoring approach that we have developed. Although we developed our programme for medical students, the issues experienced by our students are not unique to medicine, and we would expect the programme to be of benefit across a range of academic disciplines.

## Supplementary Information

Supplementary Material 1.

## Data Availability

The data that support the findings of this study are not openly available due to reasons of sensitivity.
