# Supplementary figures and images for: Improving medical students’ learning strategies, management of workload and wellbeing: a mixed methods case study in undergraduate medical education
Source: BMC Med Educ. 2025 Apr 24;25:606. doi: 10.1186/s12909-025-07118-6 (PMC12023436; doi:10.1186/s12909-025-07118-6)

## Supplementary Material- Survey Questions


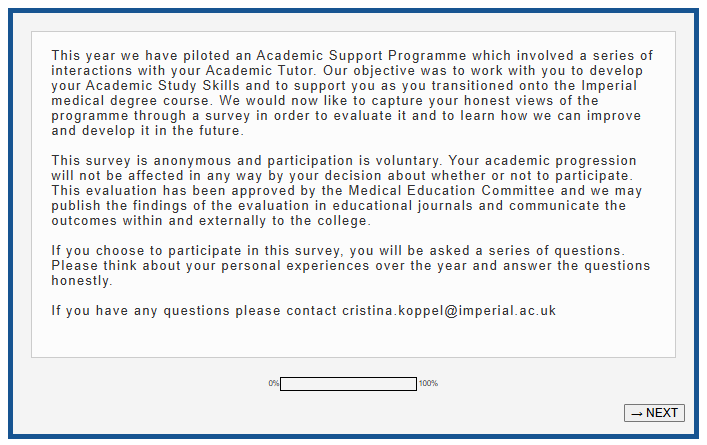


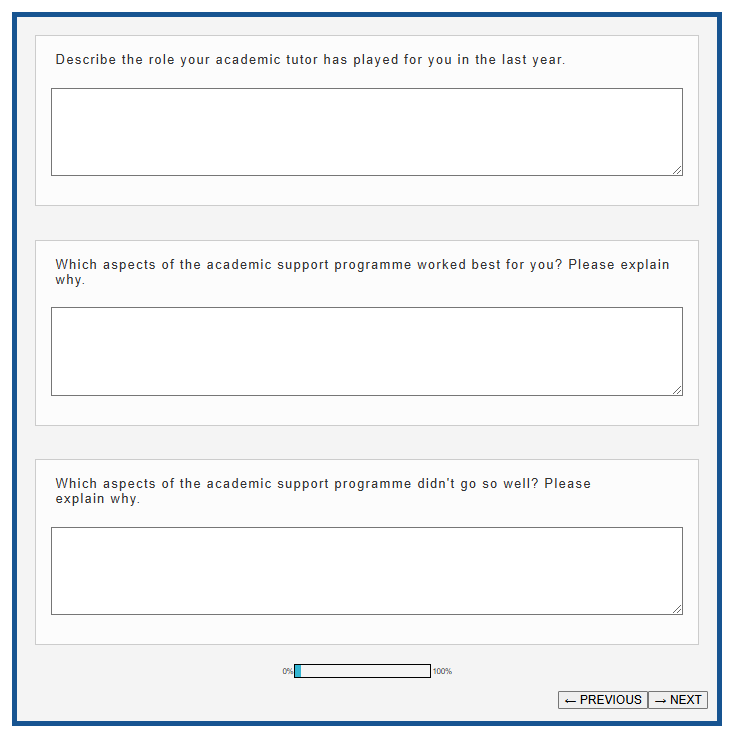


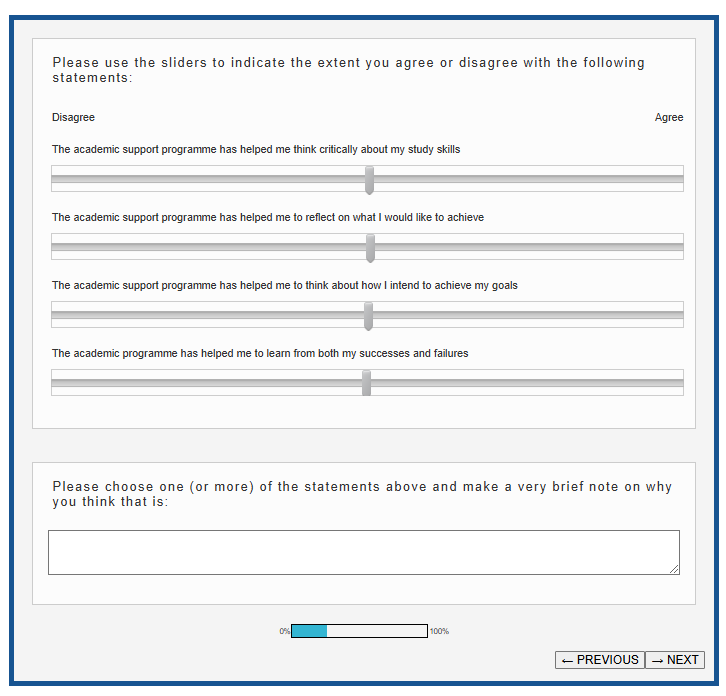


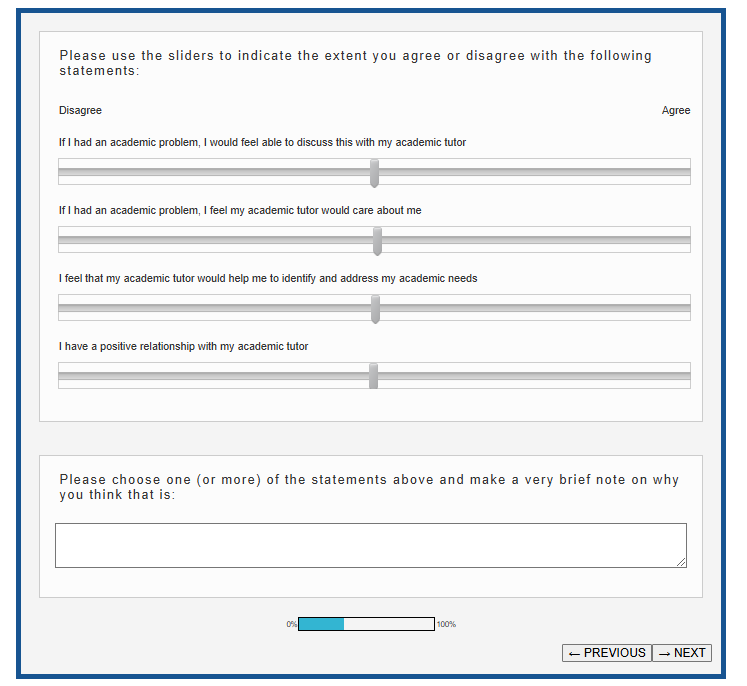

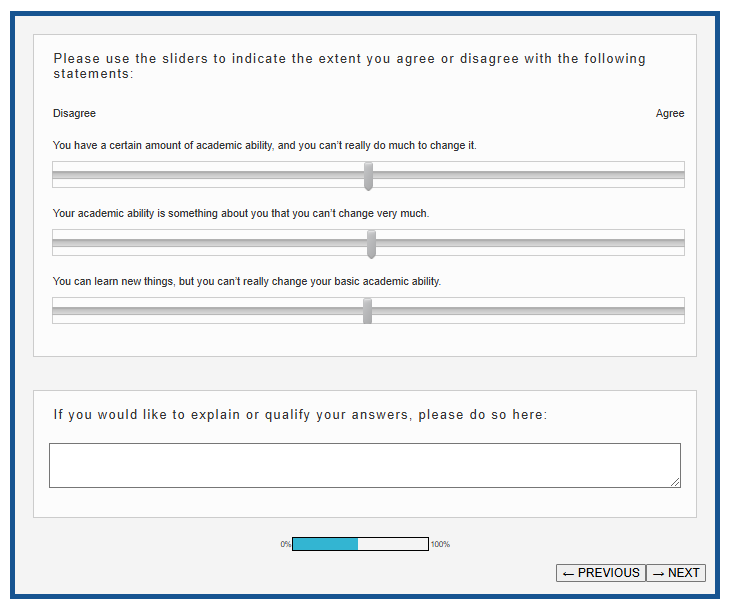

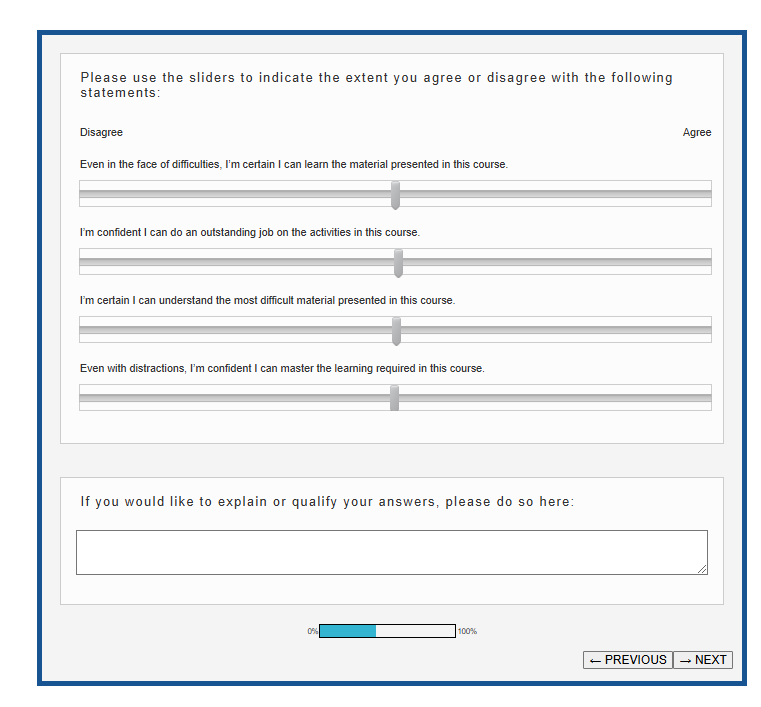

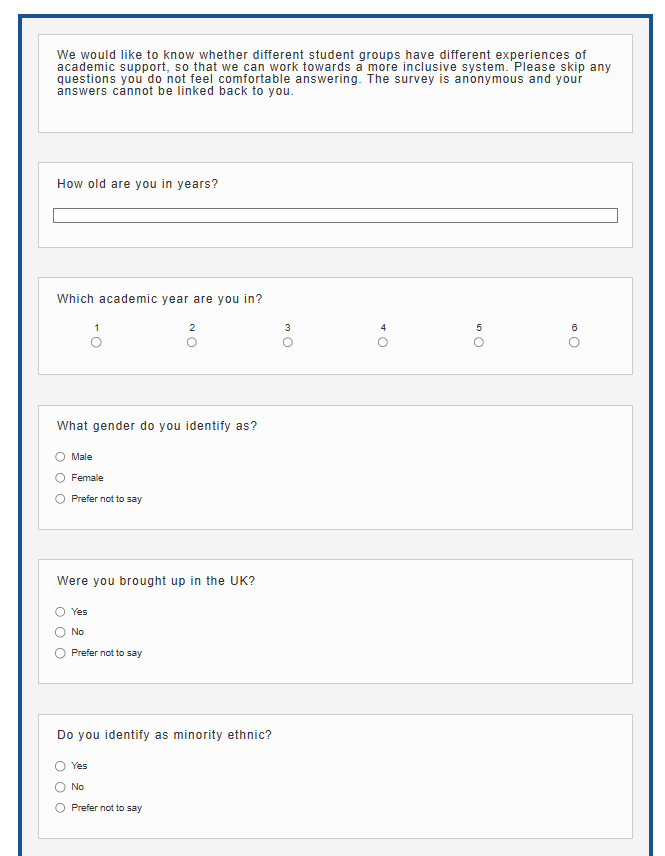

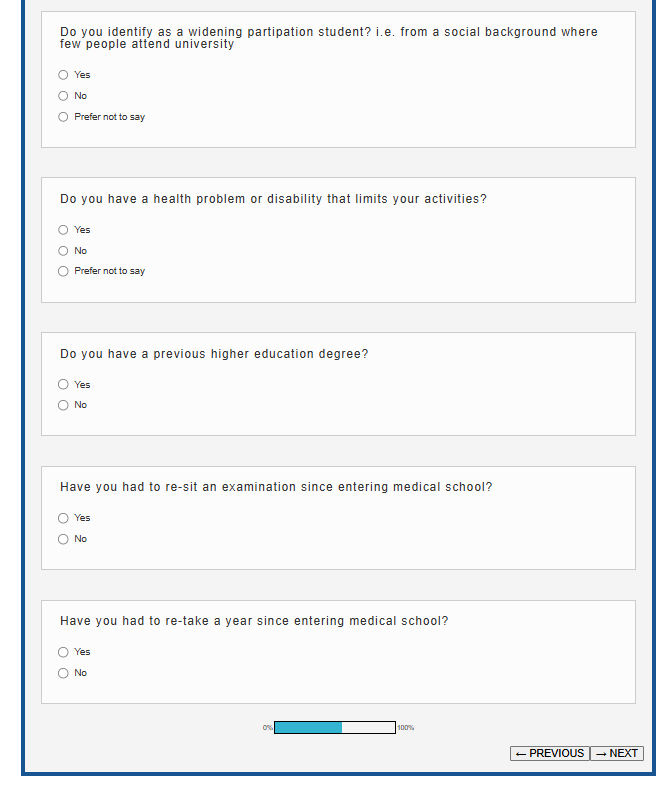

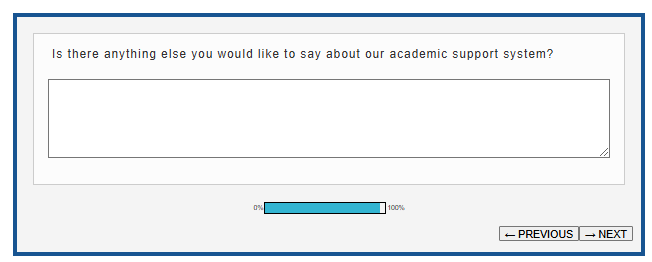

Supplement: Supplementary file 1 — Supplementary Material 1. [file 12909_2025_7118_MOESM1_ESM.docx]
